# Core microbiota of wheat rhizosphere under Upper Indo-Gangetic plains and their response to soil physicochemical properties

**DOI:** 10.3389/fpls.2023.1186162

**Published:** 2023-05-15

**Authors:** Murugan Kumar, Waquar Akhter Ansari, Mohammad Tarique Zeyad, Arjun Singh, Hillol Chakdar, Adarsh Kumar, Mohammad Samir Farooqi, Anu Sharma, Sudhir Srivastava, Alok Kumar Srivastava

**Affiliations:** ^1^ ICAR-National Bureau of Agriculturally Important Microorganisms, Mau, Uttar Pradesh, India; ^2^ ICAR-Central Soil Salinity Research Institute, Regional Research Station (RRS), Lucknow, Uttar Pradesh, India; ^3^ ICAR-Indian Agricultural Statistics Research Institute, New Delhi, India

**Keywords:** core microbiota, Indo-Gangetitc plains, keystone taxa, rhizosphere, wheat

## Abstract

Wheat is widely cultivated in the Indo-Gangetic plains of India and forms the major staple food in the region. Understanding microbial community structure in wheat rhizosphere along the Indo-Gangetic plain and their association with soil properties can be an important base for developing strategies for microbial formulations. In the present study, an attempt was made to identify the core microbiota of wheat rhizosphere through a culture-independent approach. Rhizospheric soil samples were collected from 20 different sites along the upper Indo-Gangetic plains and their bacterial community composition was analyzed based on sequencing of the V3-V4 region of the 16S rRNA gene. Diversity analysis has shown significant variation in bacterial diversity among the sites. The taxonomic profile identified Proteobacteria, Chloroflexi, Actinobacteria, Bacteroidetes, Acidobacteria, Gemmatimonadetes, Planctomycetes, Verrucomicrobia, Firmicutes, and Cyanobacteria as the most dominant phyla in the wheat rhizosphere in the region. Core microbiota analysis revealed 188 taxa as core microbiota of wheat rhizosphere with eight genera recording more than 0.5% relative abundance. The order of most abundant genera in the core microbiota is *Roseiflexus*> *Flavobacterium> Gemmatimonas*> *Haliangium*> *Iamia*> *Flavisolibacter*> *Ohtaekwangia*> *Herpetosiphon*. *Flavobacterium*, *Thermomonas*, *Massilia*, Unclassified *Rhizobiaceae*, and Unclassified Crenarchaeota were identified as keystone taxa of the wheat rhizosphere. Correlation studies revealed, pH, organic carbon content, and contents of available nitrogen, phosphorus, and iron as the major factors driving bacterial diversity in the wheat rhizosphere. Redundancy analysis has shown the impact of different soil properties on the relative abundance of different genera of the core microbiota. The results of the present study can be used as a prelude to be developing microbial formulations based on core microbiota.

## Introduction

Plants depend on the rhizospheric microbiome for nutrient uptake, and environmental stress alleviation (Simonin et al., 2020; Mahmud et al., 2021; Dhondge et al., 2022). A small subset of the rhizospheric microbiome called the core microbiota constitutes a specific set of microbial communities that are consistently associated with the plant species (Shade and Handelsman, 2012). It is believed that plants recruit these core microbiota/taxa through the secretion of root exudates into the rhizosphere (Zhalnina et al., 2018). The core microbiome is the result of the evolutionary process, such that their recruitment in the rhizosphere is beneficial to both plants and associated microbiota (Lemanceau et al., 2017). Many rhizosphere microbiome studies have shown a stronger influence of soil properties on rhizosphere microbiome, yet some findings suggest that the core root microbiome of terrestrial plants has co-evolved with their hosts in evolutionary history (Yeoh et al., 2017). Members of the core microbiota are believed to play important role in plants’ fitness, stability, and health (Shade and Stopnisek, 2019). Studies on core microbiota in rice and maize on a continental scale have shown that core microbiota are associated with multinutrient cycling in the soil and maintain complex connections among the bacterial taxa in the rhizosphere (Jiao et al., 2019). Core microbiota analyses in a restored ex-arable land have shown that the abundance of core microbiota is positively correlated with functional stability of the soil microbiome (Jiao et al., 2022). Genus, *Sphingomonas* and members of *Burkholderiaceae* were identified as core microbiota in the rhizosphere of cadmium accumulator plants. The core microbiota is believed to play important role in elevating cadmium tolerance to accumulators and influencing microbial community structure. Synthetic microbial communities comprising core microbiota when inoculated to accumulators under cadmium stress improved the overall performance (Luo et al., 2022). Keystone taxa is another concept that developed in the last decade (Agler et al., 2016; Jiang et al., 2017: Huang et al., 2019). They are defined as the taxa that are highly associated with other members of the community with significant influence on them. In agricultural system keystone taxa have been identified for a few crop species (Jiang et al., 2017; Wang et al., 2017; Huang et al., 2019; Zheng et al., 2021; Bez et al., 2023). Nine taxa were identified as keystone taxa in the rhizosphere of tobacco plants in soils suppressive for wilt caused by *Ralstonia solanacearum*. Collective abundance of these nine taxa had a significant negative correlation with abundance of *Ralstonia* (Zheng et al., 2021). Understanding the core microbiota and keystone taxa in the rhizosphere allows us to fix targets for manipulation in the region to achieve the plant’s potential growth and yield.

Wheat is an important food crop in more than 40 countries contributing to the staple needs of one-third of the global population (Kavamura et al., 2021; Nahid et al., 2022). It is widely cultivated in India with a total area under cultivation of 30.31 million ha, production of over 100 million tonnes, and productivity of 3314 kg per ha. In India, the Indo-Gangetic plains (IGP) is the major wheat growing area with a 61% share in area and 67% share in production (https://eands.dacnet.nic.in/). Challenges faced by wheat cultivation in the IGP are nutrient deficiencies, micronutrients especially, terminal heat due to climate change, deteriorated soil health, and widespread infestation of pests and diseases. Due to these challenges, productivity has got either stagnated or declined in the recent decade (Bhatt et al., 2021).

Microbe-based technologies are the way forward to mitigate these challenges. During the last few decades, several microbe-based strategies have been tried and reported for wheat cultivation in the area (Mäder et al., 2011; Kumar et al., 2017; Meena et al., 2018; Chandra and Sharma, 2021). But to succeed at a higher level it is necessary to understand the core microbiota and keystone taxa of the host plants. Understanding core microbiota and keystone taxa assists in the identification of key members of the rhizosphere microbial community that supports and sustains plant growth and yield. The core microbiota of host plants surveyed during a particular plant age varies with geographical location. Hence it is necessary to identify the composition of core microbiota in the study area to optimally manipulate them for the benefit of the host plants.

Identifying core microbiota in the rhizosphere of host plants and their response to soil physicochemical properties allows us to explore approaches for the manipulation of core microbiota for improved plant growth and yield. To the best of our knowledge, there is not a single study that focuses on understanding core microbiota and keystone taxa in the wheat rhizosphere in India and how they are influenced by different soil physicochemical properties. Our study is focused on i) identifying the core bacterial microbiota and keystone taxa of wheat rhizosphere in the Indo-Gangetic plains, India and ii) the influence of soil physicochemical properties on core microbiota and overall diversity of wheat rhizosphere.

## Materials and methods

### Sample collection

Wheat rhizospheric soil samples were collected from 20 different locations in Uttar Pradesh, India, aligning along the Upper Indo-Gangetic plains of India. Details of the sites are presented in **Table 1**. Soil samples were collected from the rhizosphere of 45-50 days old seedlings during January 2022. Samples were collected in triplicates from each location by following the standard sampling strategies. Briefly, for each replication, five different plants from sites covering the entire field were uprooted, and rhizosphere soils were collected and pooled to make a composite sample in a sterile polybag. Samples were brought to the laboratory in a cool box (4°C) and processed immediately. After sieving through a 2.0-mm sieve to remove stones and plant root debris, a part of the soil sample was used for soil physicochemical analysis. The remaining part was stored at -20°C until further usage for DNA extraction.

**Table 1:** Details of samples, with sample number, GPS coordinates, and detailed address.

S. No	GPS coordinates	Address
S1	28.006N, 78.108E	Anoopshahar road, Aligarh
S2	28.331N, 77.843E	Maman khurd, Bulandshahar
S3	28.925N, 77.724E	Jahidpur, Meerut
S4	29.268N, 77.989E	Miranpur, Muzaffarnagar
S5	29.274N, 78.272E	Noorpur road, Bijnor
S6	28.925N, 78.447E	Mohammadpur, Amroha
S7	28.821N, 78.696E	Mangupura, Moradabad
S8	28.449N, 79.328E	Madhav pur, Bareilly
S9	28.168N, 79.511E	Bareilly road, Budaun
S10	28.017N, 79.674E	Feelnagar, Shahjahanpur
S11	27.597N, 79.984E	Saidpur, Hardoi
S12	27.576N, 80.002E	Kapoorpur, Hardoi
S13	27.462N, 79.650E	Baddupur, Farrukhabad
S14	26.885N, 80.026E	Araul, Bilhaur, Kanpur
S 15	26.693N, 79.874E	Rasulabad, Kanpur
S16	26.786N, 79.697E	Piprauli, Auraiya
S17	26.784N, 79.190E	Mahtuwa, Etawah
S18	27.516N, 78.590E	Bawsa, Eta
S19	27.283N, 78.291E	Srinagar, Firozabad
S20	27.282N, 78.038E	Etmadpur, Agra

### Determination of soil properties

The soil pH was measured using a pH meter (EUTECH, Instruments, India), as described by Yan et al. (1996), while a conductivity meter (Labman Instruments, India) was used for the measurement of electrical conductivity according to Jackson (1973). The K_2_Cr_2_O_7_ oxidation method was used to quantify the amount of soil organic carbon (SOC) (Salam et al., 1999). Total nitrogen (N), phosphorus (P), potassium (K), iron (Fe), and sulphur (S) were estimated using the protocol as described by Bremner and Hauck, 1982; Knudsen et al., 1982; Olsen and Sommers, 1982; Hesse, 1994; Jankiewicz et al., 2002 respectively. To estimate the available N, P, K, Fe, and S, we followed the methodology of Subbiah and Asija, 1956; Olsen et al., 1954; Williams and Steinberg, 1959; Kirkbright and Sargent, 1974; Jankiewicz et al., 2002 respectively.

### Extraction of DNA, library preparation, and amplicon sequencing

Genomic DNA was isolated from the 250 mg of wheat rhizosphere soils (Jin et al., 2023) using the FastDNA™ SPIN Soil kit (MP Biomedicals, Solon, USA) as per the instructions of the manufacturer. For improving DNA yield, the initial bead beating step was extended to 15 minutes. The integrity of the DNA was checked on 0.8% (w/v) agarose gel, and to measure the DNA concentration, Qubit Fluorimeter (v.3.0) was used. To prepare the amplicon library, the V3-V4 variable region of the 16S rRNA gene was amplified using the primer pair of 341F (5′-CCTACGGGNGGCWGCAG-3′) and 806R (5′- GGACTACNNGGGTATCTAAT-3′) linked with adapter and barcode sequences (Oberpaul et al., 2020). The amplicons were checked and recovered from 2% agarose gel through purification while removing any nonspecific amplifications. Preparation of Amplicon libraries and pyrosequencing were performed by LGC Genomics GmbH, Berlin, Germany on the Illumina Miseq platform 300 bp paired-end read using the reagents and kits of the Miseq v3 system (Illumina MiSeq V3). For bioinformatic analysis the methods followed by Jin et al. (2023) was employed. Briefly the reads were sorted for barcode sequences and clipped while discarding reads with incorrect barcodes, missing barcodes, or conflicting barcode pairs. Selected reads were then subjected to the removal of adaptor remnants and primers. Forward and reverse sequences of each samples were merged to generate contigs. After generating contigs, sequences with ambiguous bases, homopolymer stretches (more than 8 bases), and sequences with low phred score (below 33) were removed. Quality-filtered sequence reads were clustered into operational taxonomic units (OTUs) at the 97% similarity threshold using the pipeline, mothur 1.35.1 (Schloss et al., 2009). The pipeline removes singleton reads, eliminates chimera, and creates OTU count tables. OTU diversity analysis was done using QIIME program version: 1.9.0 (Caporaso et al., 2011) and PAST version 4.5 (Martin-Laurent et al., 2001). Weighted PCoA plots was produced using the QIIME pipeline. Sequence data were deposited in the Sequence Read Archive (SRA) at NCBI with the project number PRJNA887236.

### Core microbiota and network analyses

To identify the core bacterial microbiota of the wheat rhizosphere, we selected the ubiquitous taxa present in all 20 sites. To elucidate microbe-microbe associations in the rhizosphere of wheat, cooccurrence networks based on the prevalence of the OTUs were identified. MetagenoNets pipeline was used in this analysis (Nagpal et al., 2020). The rare taxa from the datasets were removed by considering prevalence of the OTUs at 0.02%. SparCC value was calculated with correlation cut-off, based on Critical-R and p-value < 0.05. Network topology including nodes, edges diameter, density and average degrees were calculated. The nodes with highest betweenness centrality and node degree were selected as keystone taxa (Bez et al., 2023).

### Statistical analyses

Three replicates were used to determine the soil parameters, and the data were then subjected to a two-way analysis of variance (ANOVA) using SPSS 22.0 (SPSS, Chicago, IL, USA). All data were displayed as means ± standard error (SE) after the means were compared using Duncan’s multiple-range test and the HSD test. Using PAST, Pearson’s correlation analysis was carried out to assess the relationships between the diversity of the microbial community and the characteristics of the soil. Additionally, the multivariate relationship between the abundance of core genera and soil properties was measured using redundancy analysis (RDA) with PAST version 4.5 (Martin-Laurent et al., 2001). Before RDA, detrended correspondence analysis (DCA) with data on core genera abundance was carried out to choose between a linear or unimodal response model. The results indicated that RDA was more suitable for determining the relationship between the distribution of bacteria and soil characteristics.

## Results

### Bacterial diversity of wheat rhizosphere

To estimate the bacterial diversity of wheat rhizosphere from 20 sites, a 16S rRNA marker-based community metagenomics approach was adopted. The amplicon libraries from these sites were sequenced in the NGS platform and generated around 4.3 million reads. Quality-filtered merged reads were clustered into OTUs at 97% similarity. OTU numbers varied from 3,807 to 6,798, with a total of 25,405 OTUs in all samples. Detected OTUs were assigned to 47 phyla, 132 classes, 286 orders, 568 families, and 1209 genera. Sequences generated were sufficient to cover all the bacterial diversity expected in all the 20 sites as indicated by the rarefaction curve (**Supplementary Figure 1**).

**Figure 1:**
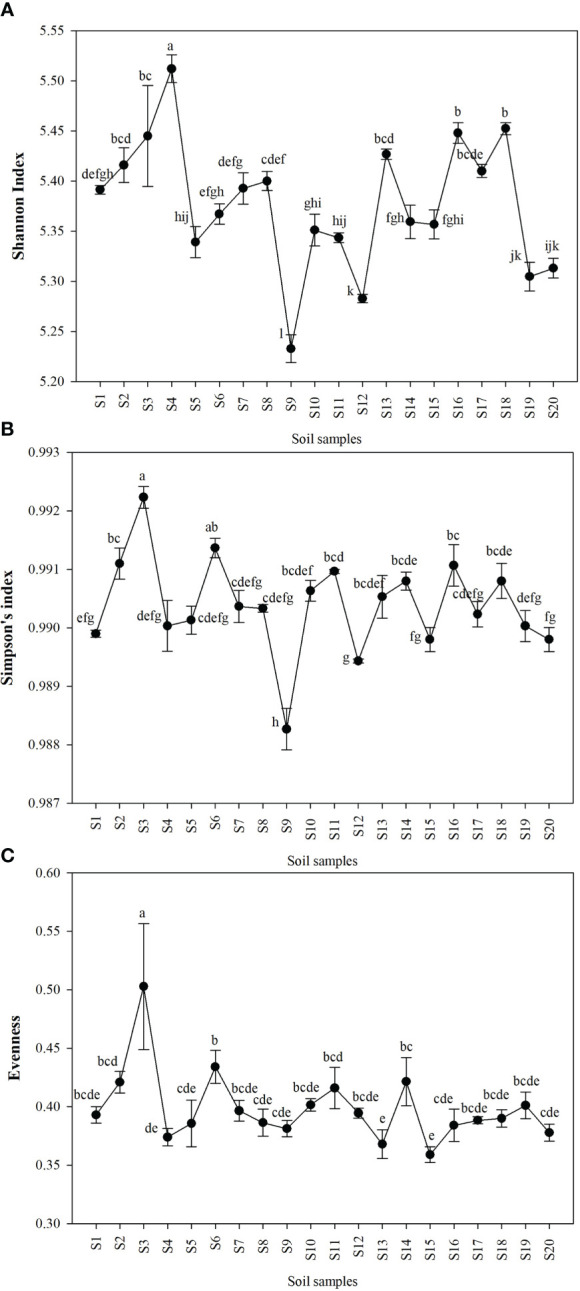
Alpha diversity of bacteria in the wheat rhizosphere in each of the sites expressed as **(A)** Shannon index **(B)** Simpson index **(C)** Evenness. The details of S1-S20 are mentioned in **Table 1**. The results are mean ± SE of triplicate measurements, means followed by the same letter are not significantly different, according to Duncan’s multiple range test.

**Figure 2:**
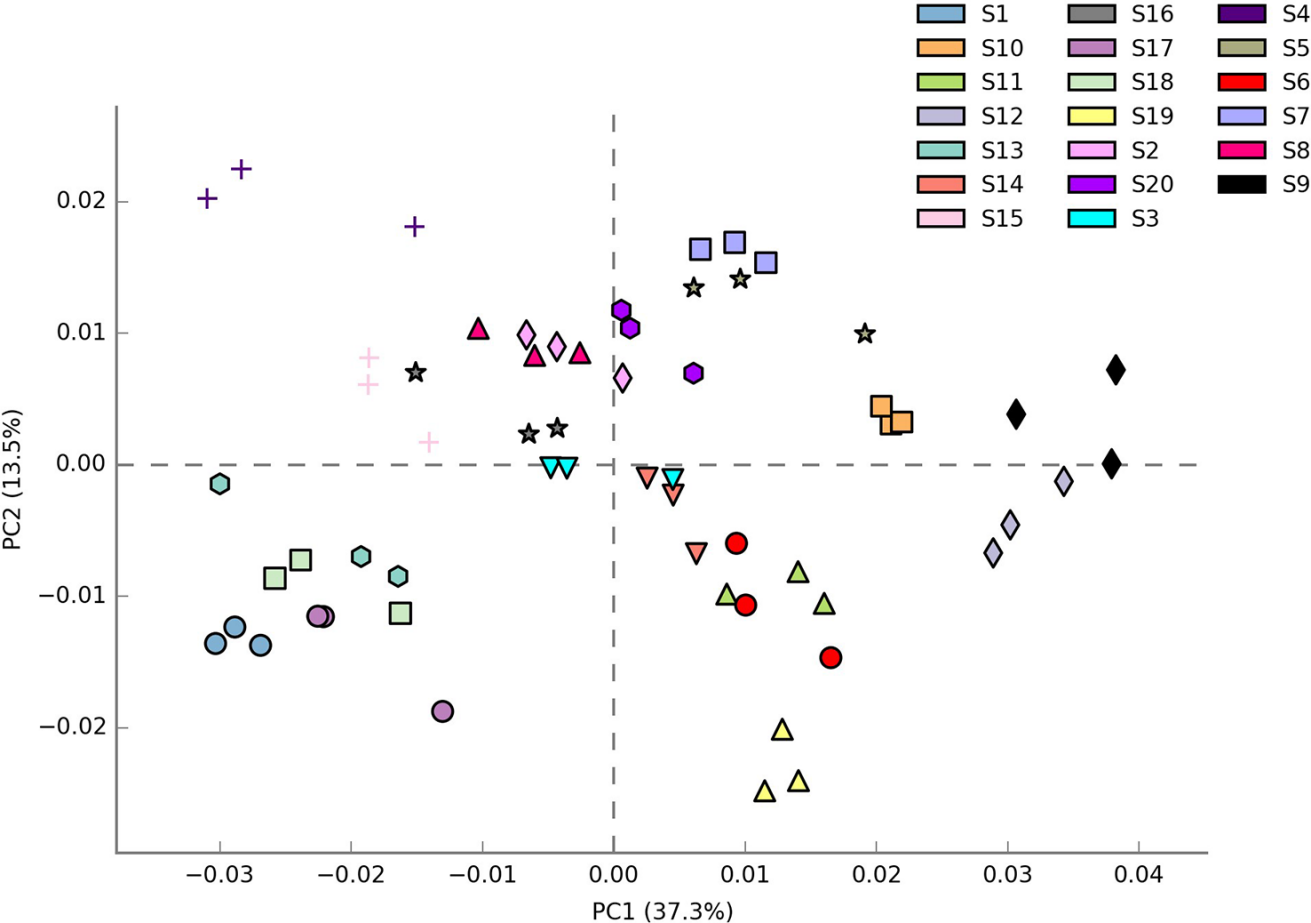
β diversity of bacteria associated with wheat rhizosphere in upper IGP as revealed by weighted unifrac distances. The details of S1-S20 are mentioned in **Table 1**.

Shannon index (H’), Simpson index (D), and Evenness (E) were calculated as a measure of the alpha diversity of bacteria in the samples. There were clear and significant differences between the sites for the indices observed (p < 0.05). Alpha diversity indices of all the 20 sites are expressed in **Figures 1A–C**. Site S3 recorded the highest Simpson index and evenness while site S4 recorded the highest Shannon index. Site S9 recorded the lowest Shannon and Simpson indices while S15 recorded the lowest evenness. Statistical differences of the rhizospheric bacterial communities of wheat rhizosphere along the Upper Indo Gangetic plain among 20 sites were estimated by principal coordinate analysis (PCoA). Overall, the first two principal coordinates explained 37.3% and 13.5% of the variation in the compositional data. There was clear differences among the sites with respect to bacterial community composition. Four distinct groups were observed *viz*., S1, S13, S17, S18 (group 1), S2, S3, S4, S8, S15, S16 (group 2), S5, S7, S9, S10, S20 (group 3) and S6, S11, S12, S14, S19 (group 4) (**Figure 2**).

### Composition of soil bacterial community

**Figure 3:**
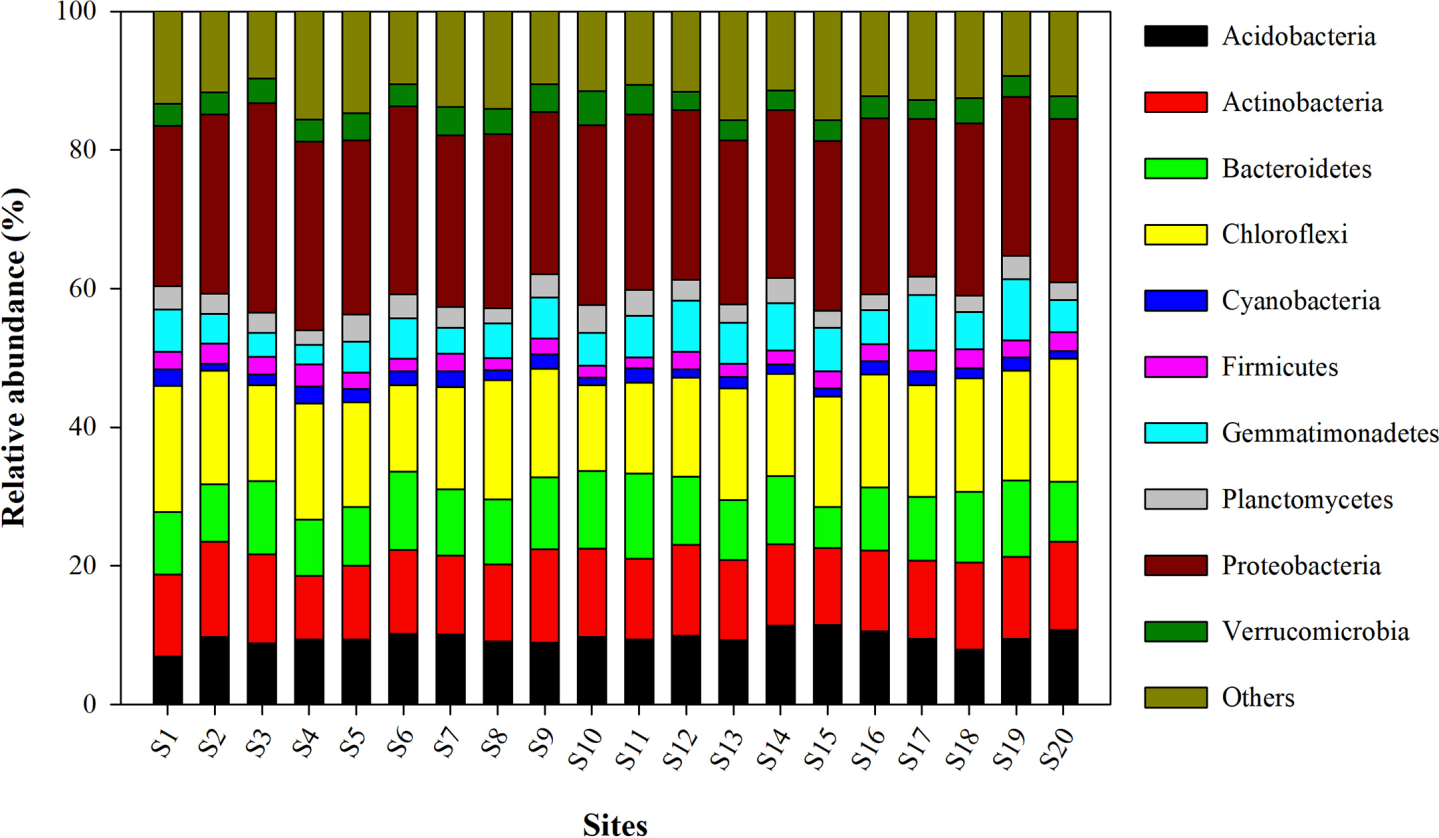
Relative abundance of different bacterial phyla observed in wheat rhizosphere across upper IGP. The details of S1-S20 are mentioned in **Table 1**.

The relative abundance of the microbial phyla in all 20 sites is presented in **Figure 3**. Among them, the most abundant bacterial phylum included Proteobacteria, Chloroflexi, Actinobacteria, Bacteroidetes, Acidobacteria, Gemmatimonadetes, Planctomycetes, Verrucomicrobia, Firmicutes, and Cyanobacteria. The mean relative abundance of Proteobacteria was found to be highest in the site S3 (30.24 ± 0.94%) and was lowest in site S17 (22.71 ± 0.81%). Site S1 (18.19 ± 0.41), and S10 (12.41 ± 0.24%) recorded respectively the highest and lowest relative abundance of Chloroflexi.

### Core microbiota and keystone taxa of wheat rhizosphere

**Figure 4:**
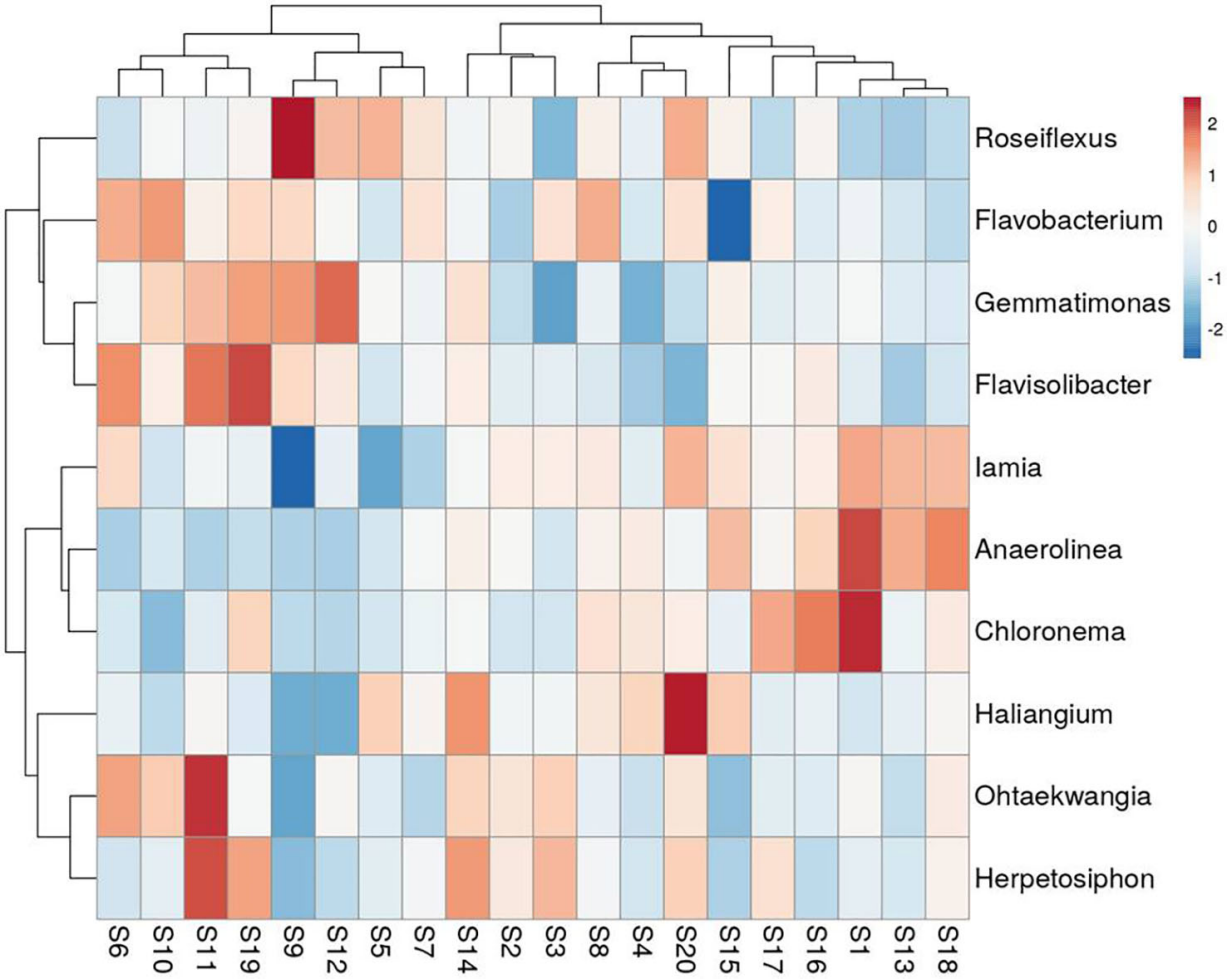
Heat-map showing the relative abundance of top 10 genera within core microbiota of wheat rhizosphere across upper IGP. The details of S1-S20 are mentioned in **Table 1**.

A total of 188 taxa were identified as core microbiota which accounts for 15.55% of all the taxa. The composition of the core genera of the wheat rhizosphere in all 20 sites is shown in **Figure 4** (top 10 genera) and **Supplementary Table 1** (top 20 genera). Of the core bacterial genera, the most abundant genera associated with wheat rhizosphere (relative abundance >0.5%) were *Roseiflexus*, *Flavobacterium*, *Gemmatimonas*, *Haliangium*, *Iamia*, *Flavisolibacter*, *Ohtaekwangia*, and *Herpetosiphon*. Cooccurrence analysis resulted in a network with 272 nodes and 1779 edges (**Supplementary Figure 2**). Top five nodes with highest betweenness centrality and degree, *viz*., *Flavobacterium*, *Thermomonas*, *Massilia*, Unclassified *Rhizobiaceae* and Unclassified Crenarchaeota were identified as keystone taxa.

### Soil physicochemical properties

Results on soil physicochemical characterization are listed in **Table 2**. To assess the soil nutrients’ diversity among the samples collected from upper Indo Gangetic plains, 13 soil physicochemical parameters were studied, which include total N, P, K, Fe, S, available N, P, K, Fe, S, organic carbon, pH, and EC. Maximum (p < 0.05) total N, P, K, Fe, and S, were recorded in sites S17, S5, S8, S18, and S4 respectively, while they were recorded lowest in sites S1, S2, S17, S6, and S15 respectively. Similarly, available N, P, K, Fe, and S, were recorded significantly (p < 0.05) highest in S15, S15, S20, S7, and S1 respectively, while they were estimated lowest in S8, S16, S5, S8, and S5 respectively. Organic carbon content was recorded highest in S16 and lowest in S13. EC and pH were found significantly different among the samples with EC ranging from 80.2 μm cm^-1^ (S14) to 298.7 μm cm^-1^ (S1). The soil pH ranged from 6.82 (S8) to 7.83 (S15).

**Table 2:** Soil physiochemical properties of all 20 samples.

Sample	EC (µS/cm)	pH	Total N (mg/kg)	Available N (mg/kg)	Total K (mg/kg)	Available K (mg/kg)	Total S (mg/kg)	Available S (mg/kg)	Available P (mg/kg)	Total P (mg/kg)	Available Fe (mg/kg)	Total Fe (mg/kg)	Organic carbon (%)
S1	298.7±5.32	7.33±0.09	1708±21.21	154±2.82	3729.4±36.52	187.2±3.86	2042.8±74.07	1257.2±37.34	50.4±2.55	438±3.29	4.18±0.12	132.5±6.23	0.485±0.02
S2	190.8±9.05	6.98±0.03	3136±31.58	86.8±3.20	3171.6±80.88	80.6±2.51	3471.4±122.23	659.4±8.66	50.4±2.16	158±2.35	3.08±0.08	165.4±5.33	0.293±0.01
S3	129.2±2.73	6.88±0.13	3388±11.31	305.2±8.39	4687.3±107.65	121.7±3.15	2233.3±47.76	728.1±10.90	57.4±2.54	478±19.79	6.70±0.23	131.8±8.25	0.659±0.00
S4	125.2±3.20	7.44±0.16	4256±39.59	137.2±4.33	3003.1±42.97	52.6±1.22	3857.1±67.34	506.9±7.06	49±1.88	498±12.72	4.00±0.19	200.2±17.6	0.414±0.00
S5	139.7±3.91	7.27±0.11	3332±28.75	210±9.89	5108.4±60.14	33.3±1.08	2790.5±108.17	280.5±2.61	58.8±1.50	828±13.19	5.35±0.32	250.9±23.5	0.655±0.01
S6	137.3±4.10	7.35±0.18	2464±17.44	81.2±3.39	5161.1±82.05	93.0±1.42	1280.9±24.01	486.2±3.91	21±0.47	208±5.65	2.98±0.14	113.8±6.32	0.520±0.01
S7	180±2.82	7.78±0.25	3808±19.79	400.4±10.55	5634.7±98.40	61.5±1.20	2976.2±52.88	620.1±6.22	72.8±1.04	588±6.59	7.75±0.47	155.2±11.4	0.786±0.02
S8	138.6±5.37	6.82±0.19	4676±13.67	58.8±3.20	7287.3±135.46	86.2±1.96	2757±48.55	1100.9±12.24	9.8±0.09	308±7.54	1.88±0.06	239±16.2	0.143±0.01
S9	175±5.65	7.62±0.08	3472±30.16	165.2±7.73	6266.3±125.54	40.5±1.67	1828.5±18.65	980.7±6.02	25.2±0.56	608±9.89	3.73±0.22	284.1±12.9	0.220±0.03
S10	175±6.59	7.12±0.37	2548±22.62	204.4±8.76	3950.5±46.44	55.6±1.56	1376.2±24.60	474.1±3.79	64.4±0.75	198±6.12	4.85±0.24	146.3±7.48	0.627±0.04
S11	118.6±4.05	7.53±0.28	4116±40.54	142.8±3.67	2908.4±29.42	140.4±3.46	2428.5±23.83	417.1±5.22	39.2±1.31	558±7.07	3.43±0.19	126.6±5.49	0.761±0.01
S12	177.3±3.44	7.02±0.14	2576±26.39	98±1.41	7245.2±113.26	55.5±1.68	1471.4±32.72	642.5±86.99	29.4±1.22	248±6.12	3.18±0.08	132.7±8.65	0.380±0.01
S13	235±6.12	7.53±0.22	4564±53.74	260.4±7.25	6055.7±62.59	70.6±1.72	2709.5±37.02	493.3±3.89	61.6±1.13	578±10.37	5.88±0.36	174.1±11.2	0.714±0.02
S14	80.2±2.73	7.66±0.12	5068±157.92	361.2±4.33	6150.5±96.88	61.5±1.19	2561.9±29.18	426.4±5.39	66±1.31	538±6.59	7.05±0.43	126.4±9.36	0.429±0.01
S15	108.4±3.01	7.83±0.14	4872±69.29	434±4.24	6687.4±170.82	129.7±3.65	995.23±11.89	593.7±5.53	99.4±2.16	648±10.37	9.50±0.68	213.8±15.8	0.857±0.01
S16	295.2±6.50	6.88±0.24	2968±46.66	64.4±1.13	4087.3±68.52	134.3±2.47	1328.5±13.46	841.8±194.13	12.6±0.75	258±5.65	2.48±0.05	278.8±13.7	0.220±0.01
S17	112.8±3.39	7.12±0.13	5292±79.66	114.8±2.26	2592.7±63.93	81.1±1.42	1600±36.29	391.1±5.61	35±0.94	388±5.65	4.25±0.22	227.4±9.62	0.414±0.01
S18	202±5.65	7.37±0.15	2716±14.61	170.8±2.73	3076.8±43.76	217.2±6.67	1995.2±47.25	451.2±9.99	57.4±1.22	528±7.07	4.90±0.33	310.5±22.6	0.586±0.03
S19	168.9±4.19	7.09±0.27	5124±57.98	103.6±1.69	3076.8±52.72	211.4±5.37	1642.8±27.74	888.5±12.98	33.6±0.75	348±8.01	4.05±0.31	150.7±7.86	0.386±0.01
S20	152.4±3.11	6.84±0.07	4452±70.71	64.4±1.13	2634.7±53.61	296.3±7.68	3709.5±57.75	816.3±16.19	19.6±0.75	358±7.54	2.93±0.16	217.7±16.9	0.220±0.02

Data are expressed as means ± standard error.

### Relationship between soil parameters and microbial community composition

**Figure 5:**
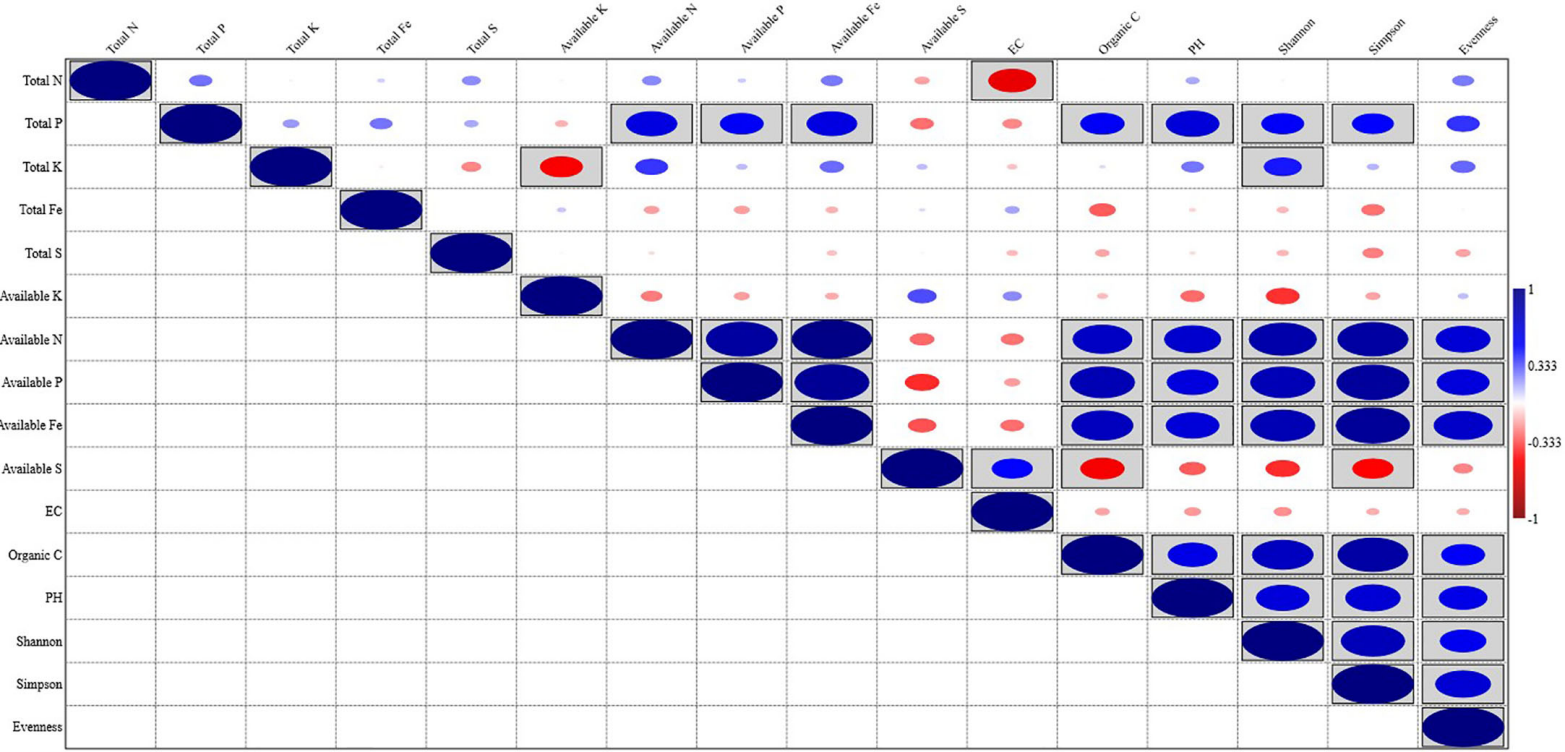
Pearson correlations between soil physicochemical properties and bacterial diversity of wheat rhizosphere.

**Figure 6:**
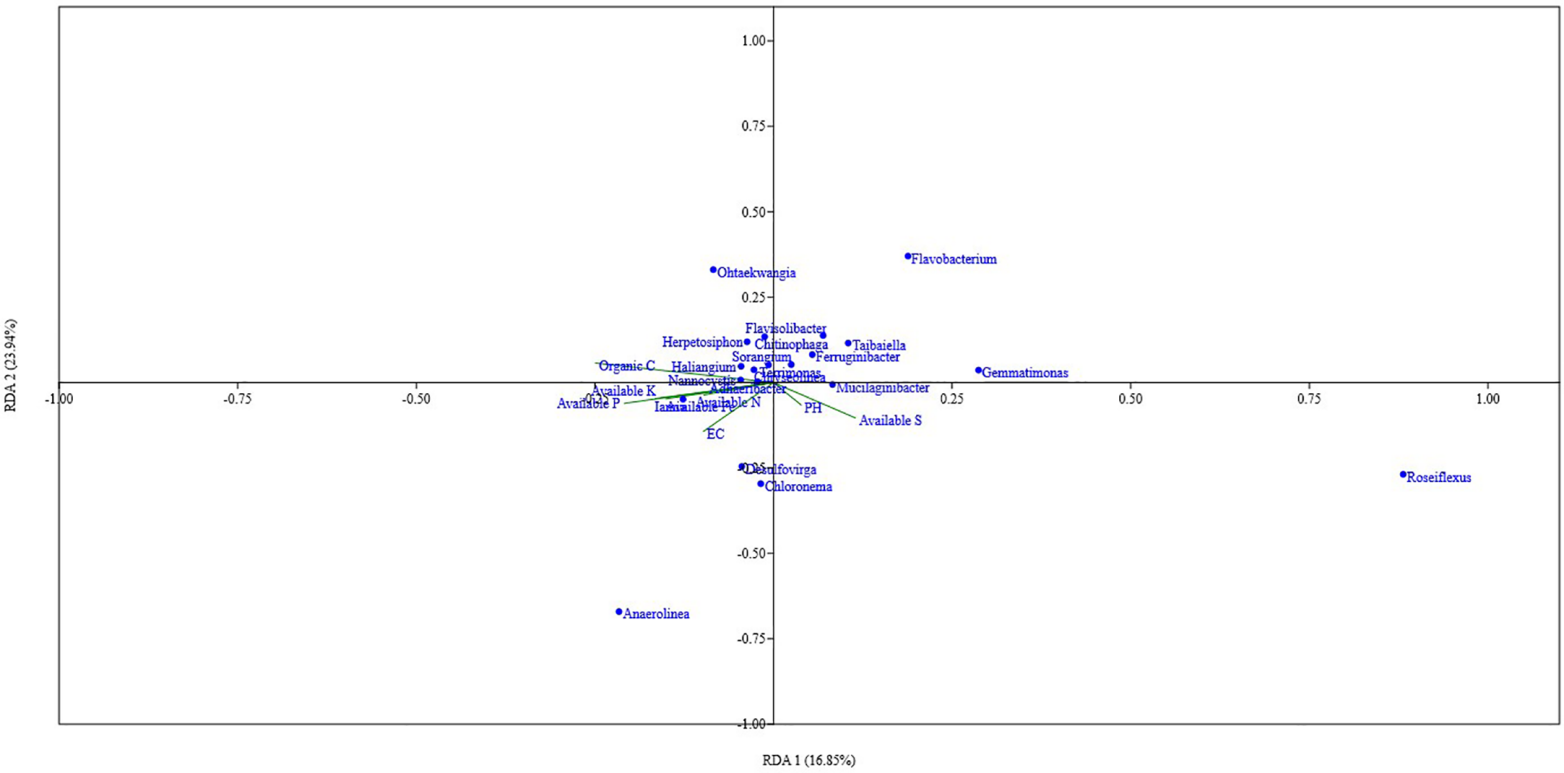
RDA analysis showing the relation between the top 20 taxa of core microbiota of wheat rhizosphere in upper IGP and soil physicochemical properties.

Organic carbon content, pH, available N, P, and Fe showed a significant positive correlation with the Shannon index, Simpson index, and Evenness. Soil parameters like Total N, P, K, Fe, S, available K, and EC did show different levels of correlation with the Shannon index, Simpson index, and Evenness; Total P showed a significant positive correlation with the Shannon index, and Simpson index, while Total K showed a significant positive correlation with Shannon index and available S showed significant negative correlation with Simpson index (**Figure 5**). Out of the total 13 soil parameters analyzed, 7 parameters showed a significant positive correlation with Shannon index, 6 parameters showed a significant positive correlation with Simpson index, and 5 parameters showed a significant positive correlation with Evenness. RDA analysis was performed to relate the abundance of the top 20 bacterial genera with soil properties in the rhizosphere of wheat collected from 20 different sites (**Figure 6**). More than 40% of the variance of bacterial community could be explained according to the 2 canonical axes, RDA 1 and RDA 2 that accounted for 16.85% and 23.94% of the variation, respectively. Most of the bacterial genera and soil nutrients factors (available P, K, Fe, N, and OC) were shown on the left side of the RDA ordination diagram, which suggested that many bacterial genera, were positively affected by the soil properties. The genera, *Haliangium*, *Iamia*, *Chitinophaga*, *Herpetosiphon*, *Terrimonas*, *Chryseolinea*, *Nannocystis*, *Ohtaekwangia*, *Adhaeribacter*, *Anaerolinea*, *Desulfovirga*, and *Chloronema* were positively influenced by OC and available P, K, Fe, and N while negatively influenced by available S. The genera *Flavisolibacter*, *Taibaiella*, *Ferruginibacter*, *Flavobacterium*, *Gemmatimonas*, *Sorangium*, *Mucilaginibacter*, and *Roseiflexus* were positively influenced by available S while negatively influenced by OC and available P, K, Fe, and N.

## Discussion

Soil microbial communities play an important role in plant growth, by assisting in nutrient uptake, production of growth hormones, transformation of nutrients, and alleviation of biotic and abiotic stresses. In addition, they are known to be actively involved in maintaining the ecological functions of certain ecosystems (Simonin et al., 2020; Mahmud et al., 2021; Dhondge et al., 2022). The advent of cost-effective, high-throughput sequencing technologies and analysis systems in the last two decades led to the expansion of our knowledge of the role of microbial communities in various ecosystems. The importance of plant-associated microbiomes has been reported in several studies (Hamonts et al., 2018; Jiao et al., 2019; Shade and Stopnisek, 2019; Mahmud et al., 2021; Dhondge et al., 2022). Microbiome manipulation for improvement in crop yield through nutrient management, pest and disease management, and abiotic stress alleviation is currently an evolving area of research. The Indo-Gangetic plains of India is a major wheat growing region in the country and has a vast diversity in edaphic factors, cropping systems, and agricultural practices. Green practices, like the use of biofertilizers and biopesticides due to their inherent beneficial properties, have been tried for wheat cultivation in the area by different groups (Mäder et al., 2011; Kumar et al., 2017; Meena et al., 2018; Chandra and Sharma, 2021). Manipulation of rhizosphere microbiome or microbiome engineering can be an addition to the long list of green practices. It is necessary to understand the composition of core microbiota so that target of manipulation can be fixed. For devising an effective manipulation strategy, in addition to knowledge of core microbiota composition, understanding the interactions among them and with other environmental factors is also necessary, as it is well known that soil environmental factors drive the microbial community and vice versa.

### Bacterial diversity and community composition of wheat rhizosphere under IGP

In this study, we investigated the bacterial communities associated with wheat rhizosphere, using samples collected from 20 sites of the Upper Indo Gangetic plain covering 19 districts of Uttar Pradesh, India. We followed 16S rRNA V3-V4 region amplicon sequencing to characterize the composition and assembly of the rhizosphere-associated bacterial community. The rarefaction curve on observed samples against the read numbers indicated sufficient sampling and sequencing were done to capture most of the bacterial diversity. Results on alpha and beta diversity revealed significant differences among the sampling sites in bacterial diversity. Sample sites differ significantly in soil physicochemical characteristics, which might explain the differences in bacterial diversity among the sites. These results corroborate the findings on the rhizosphere of wheat, maize, and rice (Rascovan et al., 2016; Jiao et al., 2019; Kuźniar et al., 2020; Rossmann et al., 2020).

Bacterial community composition analyses revealed Proteobacteria as the most dominant phyla in all the twenty sites. Proteobacteria is a common inhabitant in agricultural soils with many studies reporting them as dominant phyla in the rhizosphere (Rascovan et al., 2016; Jiao et al., 2019; Bziuk et al., 2021) and highlighting their role in biogeochemical cycles of nitrogen and carbon (Jiang et al., 2022). The other major phyla identified in the wheat rhizosphere under present study are Chloroflexi, Actinobacteria, Acidobacteria, Bacteroidetes, Gemmatimonadetes, Verrucomicrobia, Planctomyces, Firmicutes, Cyanobacteria, and Nitrospirae. Most of these phyla have previously been reported in the rhizosphere of different crops through various studies (Choudhary et al., 2018; Jiao et al., 2019; Rossmann et al., 2020; Bziuk et al., 2021; Dhondge et al., 2022). For instance, a high abundance of Actinobacteria, Chloroflexi, Bacteroidetes, and Firmicutes has been observed in the wheat rhizosphere (Rascovan et al., 2016; Rossmann et al., 2020).

### Core microbiota, keystone taxa and their potential role in plant growth promotion

Core microbiota analyses in the present study identified, *Roseiflexus*, *Flavobacterium*, *Gemmatimonas*, *Haliangium*, *Iamia*, *Flavisolibacter*, *Ohtaekwangia*, and *Herpetosiphon* as core genera in the wheat rhizosphere and most abundant among the core taxa. *Roseiflexus* is an aerobic photosynthetic bacterium, with potential plant growth-promoting properties (Ju et al., 2020; Qu et al., 2020; Gong et al., 2021). The genera *Flavobacterium* and *Flavisolibacter* have long been known as common plant growth-promoting rhizobacteria (Mayak et al., 2004; Di Benedetto et al., 2017). *Flavobacterium* is endowed with many plant growth-promoting traits including ACC deaminase activity, solubilization of phosphate, and growth hormone production (Mayak et al., 2004; Rathi et al., 2014). A higher abundance of these bacteria in the wheat rhizosphere under the present study signifies their involvement in plant growth improvement and enhanced yield.

*Gemmatimonas* is believed to be a biocontrol agent in different crops, assist plants in the phosphorus absorption, and generally reported in higher abundance in soils with neutral to near neutral pH (DeBruyn et al., 2011; Guan et al., 2013; Yin et al., 2013; Shen et al., 2014; Gkarmiri et al., 2017; Abbas et al., 2022). This corroborates with our study as most of our sampling sites recorded a pH near neutral. The genus *Haliangium* known for producing haliangicin a compound responsible for controlling many phytopathogenic fungi has been reported as a core genus in wheat landraces (Fudou et al., 2001; Fudou et al., 2002; Rossmann et al., 2022). Landraces are generally resistant to diseases and are an important source of breeding material for resistance breeding (Wang et al., 2022; Zhang et al., 2022). A high abundance of these potential biocontrol genera *Haliangium* and *Gemmatimonas* allows for exploring them as biocontrol agents in future studies. Bacterial inoculants including biopesticides based on native microflora can be a successful strategy against the introduced ones (Herrmann and Lesueur, 2013). The genus *Ohtaekwangia* has earlier been reported in the rhizosphere of sunflower, soybean, cucumber, and turfgrass (Zhang et al., 2011; Gaggìa et al., 2013; Tejeda-Agredano et al., 2013; Tian and Gao, 2014). It was hypothesized to be involved in the breaking down of complex organic matter derived from plants (Tian and Gao, 2014). The other two genera *Iamia*, and *Herpetosiphon* with higher abundance in the present study have not been explored much for their significance in agriculture productivity. A more elaborate study must be carried out for these two genera to assign specific functions and significance in agriculture.

*Flavobacterium*, *Thermomonas*, *Massilia*, Unclassified *Rhizobiaceae* and Unclassified Crenarchaeota were identified as keystone taxa of wheat rhizosphere in the upper Indo-Gangetic plains. The subnetworks established by these taxa ensures promotion of plant health. *Flavobacterium*, in addition to possessing many plant growth promoting traits is also a potential antagonizing agent against fungi owing to their chitinolytic activity. The colonization of this genus increased in wheat rhizosphere in the presence of *Rhizoctonia solani* AG8 (Yin et al., 2021). *Thermomonas* and *Massilia* were earlier reported as the core hub taxa of wheat rhizosphere, and their abundance was not affected by agricultural practices and soil types (Simonin et al., 2020). These bacterial taxa have also been reported to regulate the microbiome networks under onset of wheat yellow mosaic disease (Wu et al., 2021a). Members of *Rhizobiaceae* play major role in improving shoot length and shoot weight due to its better gibberellic acid production ability whereas members of Crenarchaeota were reported as regulators of nitrogen cycling networks of plant rhizosphere (Singh et al., 2022). Overall, the presence of these core microbiota and keystone taxa indicates healthy resilient states of the host plants. However their functions have to be studied elaborately to elucidate their role in wheat production. It is necessary to bring representatives of these core microbiota and keystone taxa to culture and prove their role in wheat production. The functions of representative isolates of keystone OTUs have been proved experimentally in an earlier study on tomato root litter decomposition (Jin et al., 2022).

### Influence of soil physicochemical factors on microbiota

Soil type, physicochemical characteristics, and geographical location drive the microbial community composition in soil. Root exudates and other factors in the rhizosphere of plants act upon the already available microbiota in the soil and recruit and modify their rhizosphere microbiome to their benefit (Gaiero et al., 2013; Rascovan et al., 2016). Hence it is understood that soil characteristics influence the rhizospheric microbiota. Effects of soil characteristics on the rhizospheric microbiota of several crop plants have been reported time and again by various groups (Bulgarelli et al., 2012; Rascovan et al., 2016; Jiao et al., 2019; Wu et al., 2021b; Dhondge et al., 2022). Correlation analyses in the present study between diversity indices and soil physicochemical properties revealed that soil pH, organic carbon content, and available nutrients especially, nitrogen, phosphorus, and iron drive the richness and evenness of bacterial community in wheat rhizosphere under upper IGP.

Our findings corroborated with the findings of Wang et al. (2014), Rascovan et al. (2016), and Wu et al. (2021b) as they reported the impacts of pH, organic carbon content, and other soil nutrients factors on rhizospheric microbial communities. Soil pH and OC are important drivers of microbial community composition (Lauber et al., 2009; Carbonetto et al., 2014; Wu et al., 2019; Wu et al., 2021b). They regulate soil microbial communities and hence are expected to affect rhizospheric communities as well. It is evident from our studies that there is a substantial influence of available nutrients especially, N, P, and Fe on bacterial diversity and vice versa. Nitrogen is the most important primary nutrient limiting crop growth and yield which are influenced by soil microbial communities. The effect of nitrogen on rhizospheric microbial communities is well established (Li et al., 2016; Jiao et al., 2019; Wu et al., 2019; Wu et al., 2021b; Dhondge et al., 2022). A significant positive correlation between available nitrogen and diversity indices represented by richness and evenness in the present study goes along the lines of these earlier reports. Phosphorus (P), like N, is a key limiting factor in crop growth; its critical level in soil determines the ability of crop plants to utilize available P to improve their yield. The effect of available P on rhizosphere microbial communities and the other way around has been reported in different crops (Lang et al., 2018; Zhuang et al., 2020). A positive correlation of available P on bacterial richness and evenness in the wheat rhizosphere under the study region is following earlier reports on wheat as well as on other crops (Rascovan et al., 2016; Wang et al., 2022; Wu et al., 2021b). Similarly, a significant correlation between available iron content and bacterial diversity indices is as per earlier studies (Wu et al., 2021b). Manipulation of root microbiomes for enhanced crop productivity is already an emerging area (Coleman-Derr and Tringe, 2014; Tkacz and Poole, 2015). The results of RDA which showed the relationship between the core microbiota and soil characteristics give us a scope to manipulate conditions to alter the microbiome in such a way it is beneficial to the host plants. The findings on the effects of different nutrients, pH, and OC on the individual components of core microbiota from this study can be utilized in microbiome manipulation programs and rhizospheric engineering strategies.

## Conclusions

Identification of core taxa and keystone taxa in the wheat rhizosphere under study can narrow down the focus on the identified taxa for better management of wheat cultivation. Our study has shown the prevalence of plant-beneficial bacteria like *Roseiflexus*, *Haliangium*, *Gammatimonas*, *Flavobacterium*, and *Flavisolibacter* in the core microbiota of wheat rhizosphere under the Indo-Gangetic plain region and identified *Flavobacterium*, *Thermomonas*, *Massilia*, Unclassified *Rhizobiaceae*, and Unclassified Crenarchaeota as keystone taxa. The prevalence of *Iamia* and *Herpetosiphon* in higher abundance with no known biological function in the rhizosphere emphasizes the need to study these genera in detail for their relevance in the rhizosphere. Our study also led to the understanding that pH, OC and available nutrients like N, P, and Fe drive the bacterial diversity in the rhizosphere of wheat. The study also identified factors and conditions that can be manipulated for use in microbiome manipulation strategies.

## Data availability statement

Sequence data were deposited in the Sequence Read Archive (SRA) at NCBI with the project number PRJNA887236.

## Author contributions

MK conceived the idea and planned the experiments. WA and MZ did soil sampling and performed the experiments. MK, WA, AS, MF, ASh, AK, and SS did statistical analysis, bioinformatics analysis, and interpretation of the data. AK monitored the soil physicochemical analysis. MK, WA has written the first draft of the manuscript. MK, HC, and AKS checked and improved the manuscript. All authors contributed and read the final version of the manuscript. All authors approved the submitted version.
