# Supplementary material for: Core microbiota of wheat rhizosphere under Upper Indo-Gangetic plains and their response to soil physicochemical properties
Source: Front Plant Sci. 2023 May 15;14:1186162. doi: 10.3389/fpls.2023.1186162 (PMC10226189; doi:10.3389/fpls.2023.1186162)
Supplement: Supplementary file 1 [file Table_1.docx]

**Supplementary Table 1. Composition of core genera (top 20 genera) of wheat rhizosphere across 20 sites of upper IGP**

| Genera | S1 | S2 | S3 | S4 | S5 | S6 | S7 | S8 | S9 | S10 | S11 | S12 | S13 | S14 | S15 | S16 | S17 | S18 | S19 | S20 | Mean |
| --- | --- | --- | --- | --- | --- | --- | --- | --- | --- | --- | --- | --- | --- | --- | --- | --- | --- | --- | --- | --- | --- |
| *Roseiflexus* | 5.75 | 8.70 | 4.81 | 7.64 | 11.71 | 6.25 | 9.87 | 9.10 | 14.96 | 8.44 | 8.09 | 11.46 | 5.49 | 8.22 | 8.88 | 8.75 | 5.98 | 5.91 | 8.79 | 11.73 | 8.527 |
| *Gemmatimonas* | 3.56 | 2.19 | 1.05 | 1.30 | 3.67 | 3.55 | 3.31 | 3.16 | 5.84 | 4.94 | 5.30 | 6.44 | 2.81 | 4.54 | 3.96 | 3.20 | 2.99 | 2.76 | 5.77 | 2.21 | 3.628 |
| *Haliangium* | 2.90 | 3.45 | 3.44 | 4.31 | 4.36 | 3.33 | 3.67 | 3.96 | 2.24 | 2.75 | 3.62 | 2.22 | 3.23 | 4.84 | 4.38 | 3.33 | 3.22 | 3.64 | 3.07 | 5.62 | 3.579 |
| *Flavobacterium* | 2.78 | 2.06 | 3.35 | 2.43 | 2.38 | 3.87 | 3.35 | 3.90 | 3.52 | 4.04 | 3.10 | 2.93 | 2.33 | 2.83 | 1.04 | 2.52 | 3.11 | 2.19 | 3.53 | 3.41 | 2.934 |
| *Flavisolibacter* | 1.95 | 1.96 | 2.00 | 1.60 | 1.81 | 2.95 | 2.15 | 1.87 | 2.57 | 2.32 | 3.03 | 2.37 | 1.60 | 2.30 | 2.19 | 2.33 | 2.19 | 1.80 | 3.20 | 1.46 | 2.182 |
| *Iamia* | 2.46 | 2.01 | 2.01 | 1.71 | 1.20 | 2.23 | 1.45 | 2.06 | 0.86 | 1.56 | 1.85 | 1.75 | 2.40 | 1.88 | 2.16 | 2.01 | 1.96 | 2.37 | 1.78 | 2.41 | 1.906 |
| *Ohtaekwangia* | 1.91 | 2.16 | 2.39 | 1.38 | 1.57 | 2.62 | 1.31 | 1.69 | 0.96 | 2.42 | 3.13 | 1.90 | 1.37 | 2.34 | 1.15 | 1.59 | 1.64 | 2.04 | 1.87 | 2.17 | 1.881 |
| *Anaerolinea* | 3.18 | 1.16 | 0.39 | 1.44 | 0.37 | 0.03 | 1.09 | 1.29 | 0.08 | 0.47 | 0.08 | 0.03 | 2.36 | 1.36 | 2.23 | 1.95 | 1.22 | 2.76 | 0.21 | 1.00 | 1.136 |
| *Chloronema* | 2.60 | 0.58 | 0.57 | 1.39 | 0.62 | 0.64 | 0.95 | 1.47 | 0.45 | 0.19 | 0.77 | 0.41 | 0.93 | 1.08 | 0.89 | 2.20 | 1.96 | 1.35 | 1.66 | 1.27 | 1.098 |
| *Herpetosiphon* | 0.94 | 1.20 | 1.44 | 0.81 | 0.92 | 0.79 | 1.03 | 1.03 | 0.62 | 0.94 | 1.72 | 0.75 | 0.85 | 1.53 | 0.71 | 0.75 | 1.25 | 1.11 | 1.51 | 1.36 | 1.064 |
| *Sorangium* | 0.70 | 0.57 | 0.93 | 0.65 | 1.41 | 0.82 | 1.48 | 1.03 | 1.04 | 0.77 | 1.06 | 0.59 | 0.57 | 1.23 | 1.03 | 0.70 | 0.42 | 0.90 | 0.66 | 1.13 | 0.884 |
| *Taibaiella* | 0.40 | 0.67 | 0.41 | 0.22 | 0.67 | 0.90 | 0.46 | 0.78 | 1.49 | 1.06 | 1.01 | 1.80 | 0.63 | 0.53 | 0.32 | 0.54 | 1.00 | 0.51 | 0.82 | 0.60 | 0.740 |
| *Chitinophaga* | 0.14 | 0.91 | 0.81 | 0.43 | 0.68 | 0.79 | 0.93 | 0.24 | 0.68 | 1.01 | 0.88 | 0.84 | 0.38 | 0.51 | 0.29 | 0.38 | 0.51 | 0.51 | 0.45 | 0.71 | 0.605 |
| *Terrimonas* | 0.51 | 0.48 | 0.36 | 0.40 | 0.56 | 0.64 | 0.31 | 0.63 | 0.30 | 0.76 | 0.60 | 1.02 | 0.67 | 0.59 | 0.47 | 0.62 | 0.71 | 0.54 | 0.39 | 0.45 | 0.550 |
| *Adhaeribacter* | 0.64 | 0.63 | 0.63 | 0.43 | 0.20 | 0.73 | 0.27 | 0.45 | 0.33 | 0.22 | 0.68 | 0.18 | 0.56 | 0.45 | 0.51 | 0.52 | 0.64 | 0.60 | 0.75 | 0.27 | 0.484 |
| *Chryseolinea* | 0.20 | 0.58 | 0.82 | 0.57 | 0.32 | 0.43 | 0.46 | 0.29 | 0.15 | 0.51 | 0.37 | 0.34 | 0.29 | 0.76 | 0.27 | 0.51 | 0.32 | 0.63 | 0.12 | 0.74 | 0.433 |
| *Desulfovirga* | 1.21 | 1.00 | 0.13 | 1.19 | 0.28 | 0.03 | 0.09 | 0.45 | 0.06 | 0.03 | 0.02 | 0.00 | 0.51 | 0.13 | 0.68 | 0.85 | 0.45 | 0.60 | 0.00 | 0.36 | 0.404 |
| *Nannocystis* | 0.50 | 0.38 | 0.70 | 0.27 | 0.29 | 0.33 | 0.27 | 0.48 | 0.12 | 0.48 | 0.41 | 0.09 | 0.61 | 0.67 | 0.50 | 0.34 | 0.42 | 0.45 | 0.36 | 0.71 | 0.419 |
| *Ferruginibacter* | 0.24 | 0.19 | 0.32 | 0.35 | 0.61 | 0.60 | 0.61 | 0.52 | 0.59 | 0.60 | 0.47 | 0.31 | 0.32 | 0.43 | 0.06 | 0.33 | 0.23 | 0.12 | 0.30 | 0.24 | 0.372 |
| *Mucilaginibacter* | 0.23 | 0.48 | 0.13 | 0.11 | 0.29 | 0.46 | 0.28 | 0.28 | 1.12 | 0.67 | 0.33 | 0.53 | 0.21 | 0.30 | 0.09 | 0.16 | 0.26 | 0.27 | 0.18 | 0.24 | 0.33 |
