# Supplementary material for: Core microbiota of wheat rhizosphere under Upper Indo-Gangetic plains and their response to soil physicochemical properties
Source: Front Plant Sci. 2023 May 15;14:1186162. doi: 10.3389/fpls.2023.1186162 (PMC10226189; doi:10.3389/fpls.2023.1186162)
Supplement: Supplementary file 2 [file Table_2.docx]

Supplementary Table 2 SparCC values generated for network analysis

| Node_Index | Label | Taxa | Degree | Betweenness | Coreness |
| --- | --- | --- | --- | --- | --- |
| 89 | OTU_423 | *Flavobacterium* | 25 | 1 | 9 |
| 84 | OTU_511 | *Thermomonas* | 24 | 0.887898 | 9 |
| 110 | OTU_5 | *Massilia* | 22 | 0.764495 | 9 |
| 121 | OTU_34 | Unclassified Rhizobiaceae | 21 | 0.759205 | 9 |
| 241 | OTU_191 | Unclassified Crenarchaeota | 21 | 0.685536 | 9 |
