# Supplementary figures and images for: Core microbiota of wheat rhizosphere under Upper Indo-Gangetic plains and their response to soil physicochemical properties
Source: Front Plant Sci. 2023 May 15;14:1186162. doi: 10.3389/fpls.2023.1186162 (PMC10226189; doi:10.3389/fpls.2023.1186162)

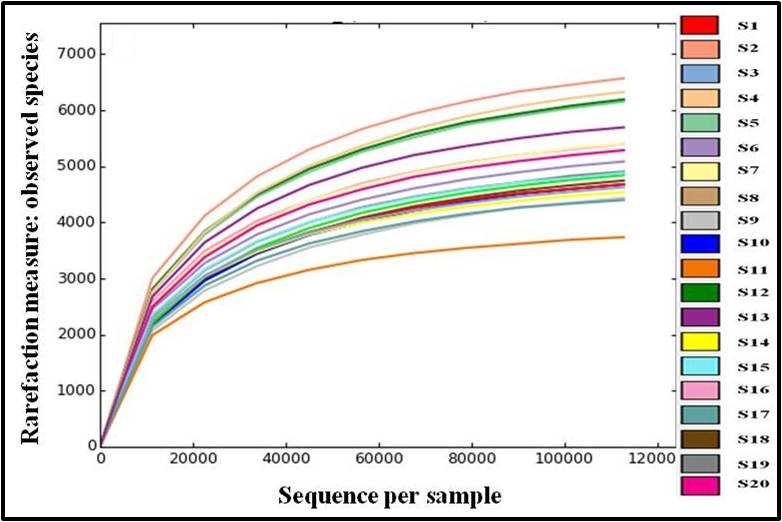

Supplement: Supplementary file 3 [file Image_1.jpeg]

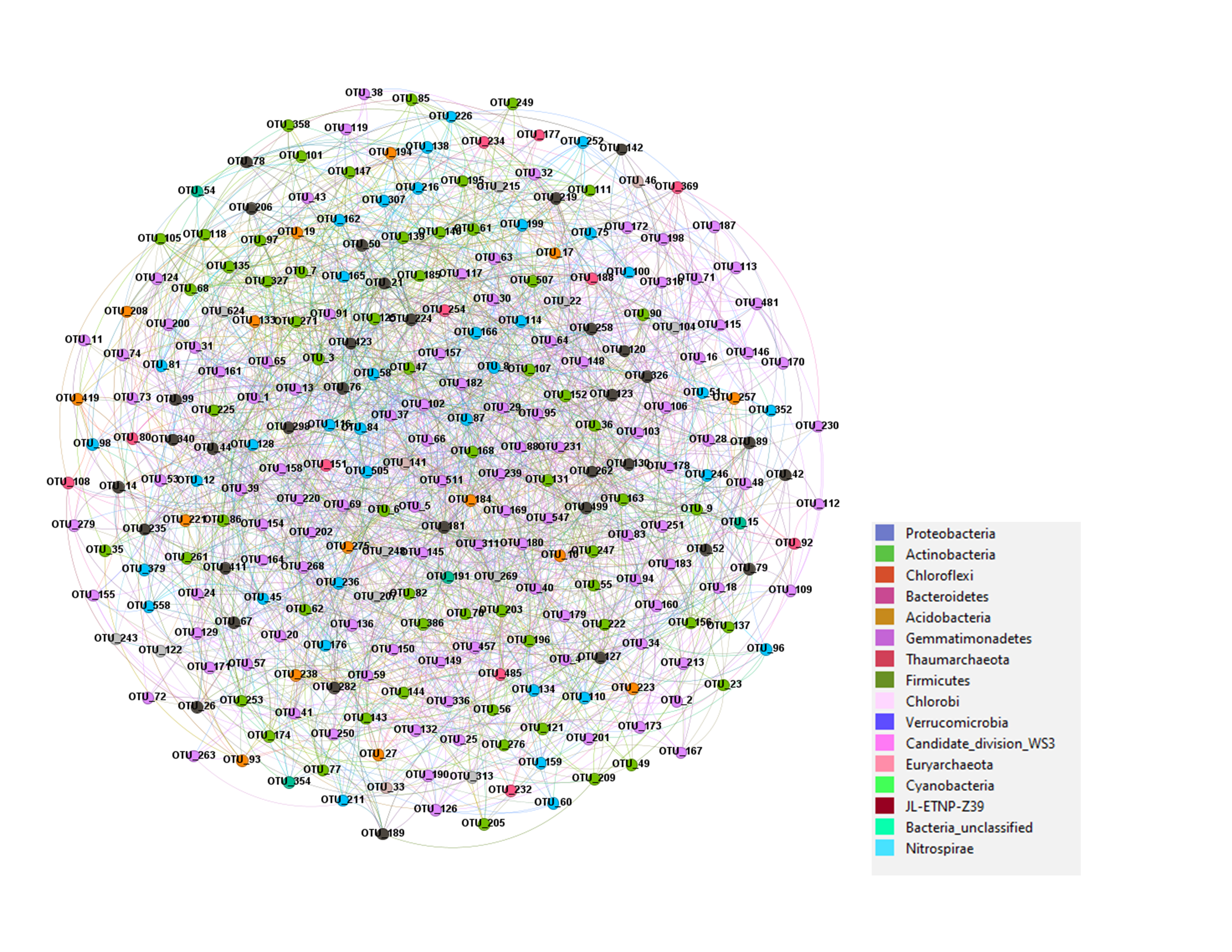

Supplement: Supplementary file 4 [file Image_2.tif]
